# Reduction of SCUBE3 by a new marine-derived asterosaponin leads to arrest of glioma cells in G1/S

**DOI:** 10.1038/s41389-020-00252-4

**Published:** 2020-08-06

**Authors:** Peng-Cheng Qiu, Yun-Yang Lu, Shan Zhang, Hua Li, Han Bao, Yu-Qiang Ji, Fei Fang, Hai-Feng Tang, Guang Cheng

**Affiliations:** 1 Institute of Materia Medica, Key Laboratory of Gastrointestinal Pharmacology of Chinese Materia Medica of the State Administration of Traditional Chinese Medicine, School of Pharmacy, Air Force Medical University, 710032 Xi’an, People’s Republic of China; 2 School of Pharmacy, Shaanxi University of Chinese Medicine, 712046 Xianyang, People’s Republic of China; 3 Central Laboratory of Xi’an No.1 Hospital, 710002 Xi’an, People’s Republic of China; 4 Department of Neurosurgery, Xijing Institute of Clinical Neuroscience, Air Force Medical University, 710032 Xi’an, People’s Republic of China

**Keywords:** CNS cancer, Drug discovery and development

## Abstract

Many saponins are characterized as exhibiting a wide spectrum of antitumor activities at low concentrations. Most of the previous studies that aimed to understand the mechanisms underlying anticancer saponins have focused on numerous classical signaling pathways. However, at the oncogene level, little is known about the action of saponins, especially asterosaponin. In this study, CN-3, a new asterosaponin isolated from the starfish *Culcita novaeguineae*, decreased the proliferation of U87 and U251 cells at low doses in a dose- and time-dependent manner. Microarray analysis revealed CN-3 significantly induced the differential expression of 661 genes that are related to its antiglioma effect in U251. Nine downregulated genes (*SCUBE3, PSD4, PGM2L1, ACSL3, PRICKLE1, ABI3BP, STON1, EDIL3,* and *KCTD12*) were selected, for further verification of their low expression. Then, shRNA transfection and high-content screening were performed and significantly decreased U251 cell proliferation rate was only observed for the SCUBE3 knockdown. qPCR confirmed SCUBE3 was highly expressed in U251 and U87 cells, and had medium expression levels in U373 cells. Real-time cellular analysis using iCELLigence demonstrated that SCUBE3 is an oncogene in U251 and U87 cells, with knockdown of SCUBE3 inhibiting U251 and U87 cell proliferation while, conversely, SCUBE3 overexpression promoted their proliferation. Afterward, SCUBE3 protein was found to have high expression in primary glioma specimens from patients examined by immunohistochemistry but low expression in normal brain. PathScan ELISA analysis in conjunction with TEM observation demonstrated that the effect of SCUBE3 knockdown in U251 does not appear to be related to the induction of apoptosis. Employing CCK-8, iCELLigence, flow cytometry, western blotting, and shRNA transfection (knockdown and overexpression) experiments, we reveal that the reduction of SCUBE3 expression, induced by CN-3, mediated both inhibition and G1/S arrest of U251 via the Akt/p-Akt/p53/p21/p27/E2F1 pathway.

## Introduction

Gliomas are the most common primary intracranial tumor, representing ~24.7% of all primary brain and other CNS tumors^1^. Glioblastoma is the most common and malignant type of glioma (~45% of all gliomas), and it is associated with a median overall survival of 15 months after resection and combined radiochemotherapy^2^, and the 5-year relative survival rate is ~5%^3,4^. Thus, there is an urgent need to find new antiglioma drugs. Natural product-derived compounds have displayed potent antitumor activities. For example, saponins, characterized by a wide spectrum of antitumor activities, are derived from some higher plants and animal sources^5,6^. Including our previous work in some cases, anticancer saponins have been isolated from *Anemone tomentosa*^7^, *Clematis argentilucida*^8^, *Anemone taipaiensis*^9–12^, *Ardisia pusilla*^13,14^, starfish *Culcita novaeguineae*^15^, and sea cucumbers^16–23^. Notably, the inhibition of cancer cell proliferation was observed in the presence of low saponin concentrations.

Oncogenes have important roles in cancer development. In light of recent advances in understanding the molecular pathogenesis of gliomas, the 2016 CNS WHO remarkably redefined diffuse gliomas based on molecular parameters and traditional histology features^4,24^. This was considered remarkable as most of the previous studies that aimed to understand the mechanisms underlying anticancer saponins had focused on the many classical signaling pathways^6^. In terms of its effects on oncogenic mechanisms, little is known about the action of saponins, especially asterosaponin. This factor has slowed down the implementation of saponin as a candidate for antiglioma therapy. SCUBE3 (signal peptide, CUB domain, and EGF-like domain-containing 3) is a member of the SCUBE family of secreted glycoproteins^25^. SCUBE3 is highly expressed in osteosarcoma and non-small cell lung cancer, and SCUBE3 knockdown has been observed to inhibit the proliferation of cancer cells^26–28^. Non-small cell lung cancer patients with low SCUBE3 expression have a significantly longer survival time^29^. However, little is known about the patterns of SCUBE3 expression and its potent mechanism in gliomas. In fact, to our best knowledge, there are no previous reports of any detrimental growth effects on glioma cell lines following SCUBE3 knockdown. In the current study, a new asterosaponin named CN-3, isolated from starfish *C novaeguineae*, inhibited glioma cell proliferation at low concentrations. Gene microarray, qPCR, high-content screening (HCS), small hairpin RNA (shRNA) transfection, flow cytometry, transmission electron microscopy (TEM), and real-time cellular analysis (RTCA) were used to determine the functional oncogene operating under CN-3 suppression of U251 and U87 cells proliferation. As a result, SCUBE3 is an oncogene for cell proliferation in U251 and U87 cells. And the further mechanism experiments revealed the reduction of SCUBE3 expression in U251 cells, mediated by CN-3, was revealed to interfere with G1/S transition via the Akt/p-Akt/p53/p21/p27/E2F1 pathway.

## Results

### The new asterosaponin isolated from starfish *Culcita novaeguineae*, CN-3, inhibits glioma cell proliferation at low concentrations

**Fig. 1:**
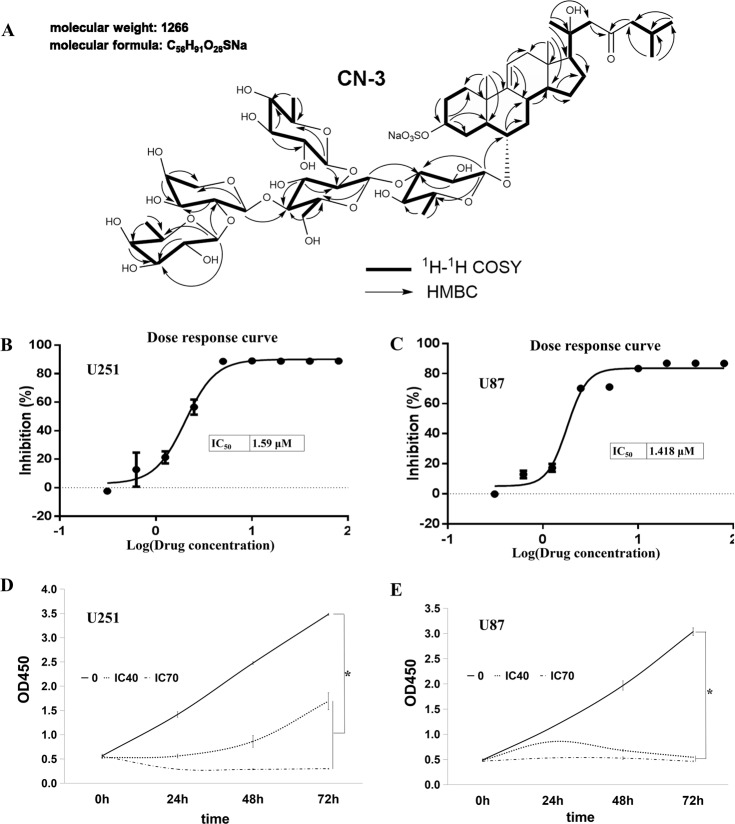
A low concentration of CN-3 inhibited glioma cell proliferation. **a** The ^1^H–^1^H COSY and key HMBC correlations of the asterosaponin CN-3; **b** IC_50_ value of 1.59 μM CN-3 for U251 cells (*n* = 3, 48 h); **c** IC_50_ value of 1.418 μM CN-3 for U87 cells (*n* = 3, 48 h); **d** IC_40_ (1.42 μM) was used as a low dose and IC_70_ (2.47 μM) was used as a high dose of CN-3 for treating U251 cells (*n* = 3, *p < 0.05 compared with normal U251 cells); **e** IC_40_ (1.34 μM) was used as a low dose and IC_70_ (2.05 μM) was used as a high dose of CN-3 for treating U87 cells (*n* = 3, *p < 0.05 compared with normal U87 cells).

The new asterosaponin CN-3 was isolated from starfish *C. novaeguineae*: sodium (20 *S*)-6*α*-*O*-{*β*-D-fucopyranosyl-(1–2)-*α*-l-arabinopyranosyl-(1-4)-[*β*-d-quinovopyranosyl-(1–2)]-*β*-d-glucopyranosyl-(1–3)-*β*-d-quinovopyranosyl}-20-hydroxy-23-oxo-5*α*-cholest-9(11)-en-3*β*-yl sulfat; m.p. 203–204 °C; [α]22D: +6°(c 0.15, MeOH); IR (KBr) *ν*_max_ 3431, 1644, 1242, 1213, 1065 cm^−1^; ^1^H-NMR and ^13^C-NMR; ESI-MS (positive ion mode): *m*/*z* 1289 [M+Na]^+^; ESI-MS (negative ion mode): *m*/*z* 1265 [M-H]^−^; HR-ESI-MS (positive ion mode): *m*/*z* 1289.5206 [M+Na]^+^(calcd for C_56_H_91_Na_2_O_28_S 1289.5213) (Table 1 and Fig. 1a). CN-3 was as white, amorphous powder, which was positive to Liebermann–Burchard and Molish tests. CN-3 inhibited glioma cell proliferation in a dose- and time-dependent manner. To evaluate the relationship between CN-3 and glioblastoma, two human glioblastoma cell lines were each treated with 0, 0.3125, 0.625, 1.25, 2.5, 5, 10, 20, 40, and 80 μg/mL of the saponin, and cell viability was examined using a CCK-8 assay after 48 h of treatment. This revealed an IC_50_ (half maximal inhibitory concentration) value of 1.59 μM (2.013 μg/mL) for U251 cells (Fig. 1b) and 1.418 μM (1.795 μg/mL) for U87 cells (Fig. 1c). Then, IC_40_ was used as a low dose and IC_70_ was used as a high dose of CN-3 for treating U251 and U87 cells. As a result, 1.42 μM (IC_40_) CN-3 suppressed U251 cell proliferation. Treatment with the low dose of 1.42 μM CN-3 resulted in a reduction in U251 cell viability from 100% to 42.5% (24 h), 37.4% (48 h), and 52.1% (72 h) (*p* < 0.05) (Fig. 1d). However, 1.34 μM (IC_40_) of CN-3 killed almost all U87 cells after 48 h (Fig. 1e). Therefore, U251 cells treated with 1.42 μM CN-3 (48 h) were selected for further experiments using microarrays.

**Table 1:** The 1H-NMR (500 MHz) and 13C-NMR (125 MHz) data of asterosaponin CN-3 from *Culcita novaeguineae* in C5D5N.

Position	δH	δC	Position	δH	δC
1	1.27, m 1.53, m	36.0	Qui I		
2	2.62,m 1.71, m	29.5	1	4.68, d, 7.2	105.1
3	4.83, m	77.6	2	3.84, m	74.2
4	3.43, br d (11.0) 1.55, m	30.7	3	3.69, m	90.4
5	1.35, m	49.3	4	3.44, t (8.8)	74.5
6	3.67, m	80.5	5	3.54, m	72.0
7	1.15, m 2.57, m	41.6	6	1.46, d (6.4)	18.4
8	1.97, m	35.3	Glc		
9	—	145.5	1	4.98, d (7.8)	103.9
10	—	38.3	2	4.14, m	81.3
11	5.13, br s	116.7	3	4.12, m	75.4
12	1.92, m 2.16, br d (16.6)	42.5	4	4.09,	80.3
13	—	41.5	5	3.97, m	76.6
14	1.18, m	53.9	6	4.62, d (12.2) 4.51, dd (13.0, 6.2)	61.9
15	1.68, m 2.12, m	23.3	Qui II		
16	1.16, m 1.68, m	25.1	1	5.14, d (6.4)	105.0
17	1.55, m	59.6	2	3.92, m	76.2
18	0.90, s	13.5	3	3.97, m	76.7
19	0.84, s	19.3	4	3.86, m	75.5
20	—	73.7	5	3.56, m	73.6
21	1.46, s	27.1	6	1.63, d (6.0)	17.9
22	2.60, ABq (15.6)	54.8	Ara		
23	—	211.5	1	4.84, d (7.2)	101.3
24	2.29, dq (15.6, 6.8)	54.0	2	4.29, m	81.7
25	2.09, m	24.4	3	4.14, m	73.3
26	0.79, d (6.6)	22.6	4	4.19, m	67.9
27	0.79, d (6.6)	22.4	5	3.59, m 4.22, m	65.6
			Fuc		
			1	4.72, d (7.8)	106.6
			2	4.26, m	73.4
			3	3.89, m	75.1
			4	3.84, m	72.5
			5	3.53, m	71.9
			6	1.32, d (6.3)	17.1

### SCUBE3 was significantly downregulated in CN-3-treated U251 cells as observed by microarray and qPCR

**Fig. 2:**
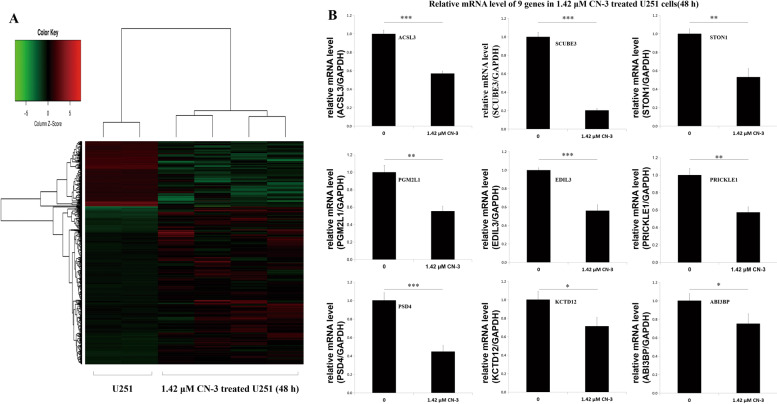
Microarray and qPCR indicate CN-3 suppressed SCUBE3 expression. **a** Between normal U251 cells (*n* = 2) and CN-3-treated U251 cells (*n* = 4), 452 genes were upregulated, and 209 genes were downregulated (GSE108343); **b** qPCR confirmed significant downregulation of nine genes (*SCUBE3, PSD4, PGM2L1, ACSL3, PRICKLE1, ABI3BP, STON1, EDIL3,* and *KCTD12*) in U251 cells treated with 1.42 μM CN-3 for 48 h (*n* = 3, *p < 0.05, **p < 0.01, and ***p < 0.001 compared with normal U251 cells).

To determine potential oncogenes associated with gliomas using CN-3, Affymetrix gene microarray analysis was carried out between untreated U251 cells and 1.42 μM CN-3-treated U251 cells (48 h) to identify genes whose expression levels were significantly different, presumably as a result of treatment. As a result, 661 genes had significantly differential expression levels, including 452 genes that were upregulated and 209 genes that were downregulated (https://www.ncbi.nlm.nih.gov/geo/query/acc.cgi?acc=GSE108343) (Fig. 2a). Nine (*SCUBE3, PSD4, PGM2L1, ACSL3, PRICKLE1, ABI3BP, STON1, EDIL3,* and *KCTD12*) of the 209 downregulated genes were selected for further verification, by qPCR, of their downregulated expression in U251 cells in response to treatment with 1.42 μM CN-3. Among the nine genes, SCUBE3 showed the greatest downregulation with the lowest relative expression level following treatment (*p* < 0.01) (Fig. 2b). The primers of the nine genes are shown in Table 2.

**Table 2:** The primers of 10 genes.

Genes	Forward	Reverse	Base pairs
*GAPDH*	TGACTTCAACAGCGACACCCA	CACCCTGTTGCTGTAGCCAAA	121
*PGM2L1*	CGAGATCGTCTTTGTTGCCGA	AGCCTCTCTGCTTGAAGTCTG	160
*EDIL3*	TGAGTGCCCAGGCGAATTTAT	ACGTGCATAGTAGGGATACCATT	157
*SCUBE3*	GCTGTGTCAACATGATGGGC	CCGCTGGATACAGGTATGCTG	86
*KCTD12*	GAGTTTTAGAAGGCTGGATGG	GTTTAAGCTACAGGCTGAGACC	179
*ACSL3*	GGATGGTTTGGCTTCAGTATT	CGATGTTGGTCTTTGGTTTCT	266
*STON1*	AGTTGGGGTCCACATCGTA	CGGCAACTCAAGGTCATTC	263
*ABI3BP*	GTTTGGGGTGTCTACTTCT	GTTTGGTGATACATTGCTG	200
*PRICKLE1*	GGAATGAATCGGTTTCTGGG	CAAACTGAGGGGTGGGAAGT	193
*PSD4*	TACGAGAAAACCCGCTACGA	CCACAGGTCCAGAGCATCAG	81

### SCUBE3 was highly expressed in U251 and U87 cells, and SCUBE3 is an oncogene for cell proliferation in U251 and U87 cells

**Fig. 3:**
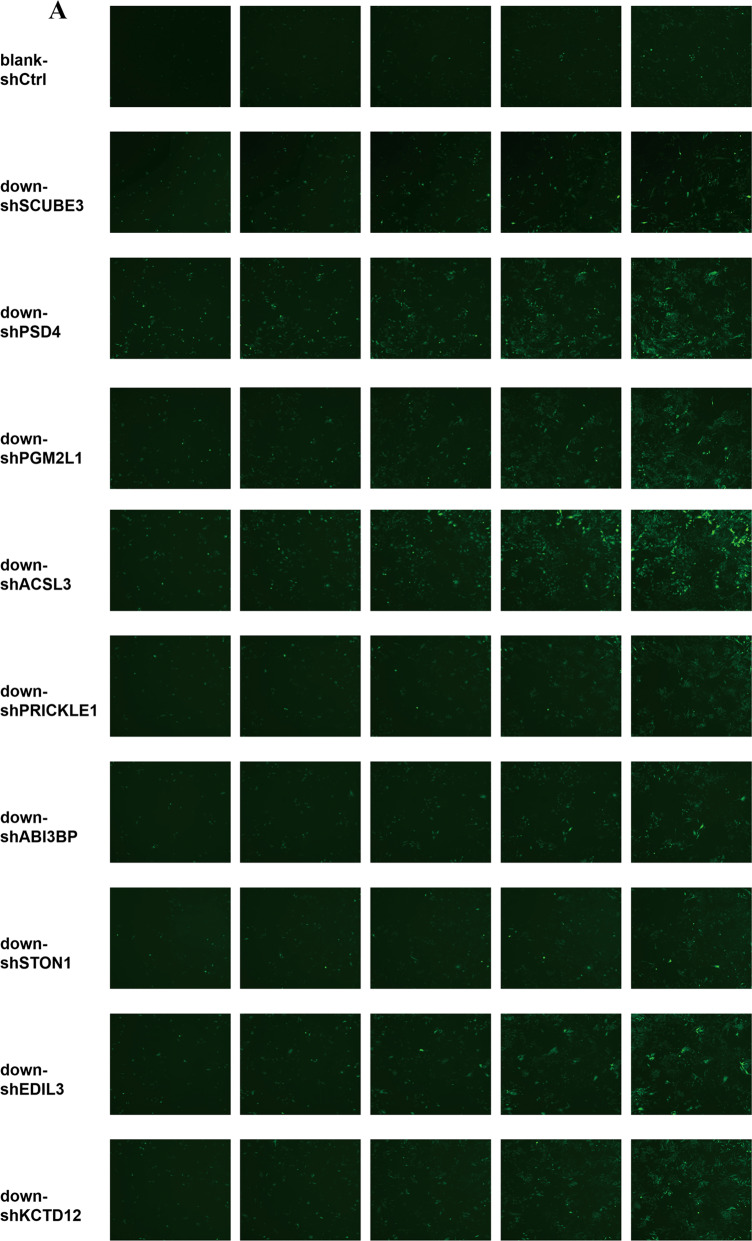

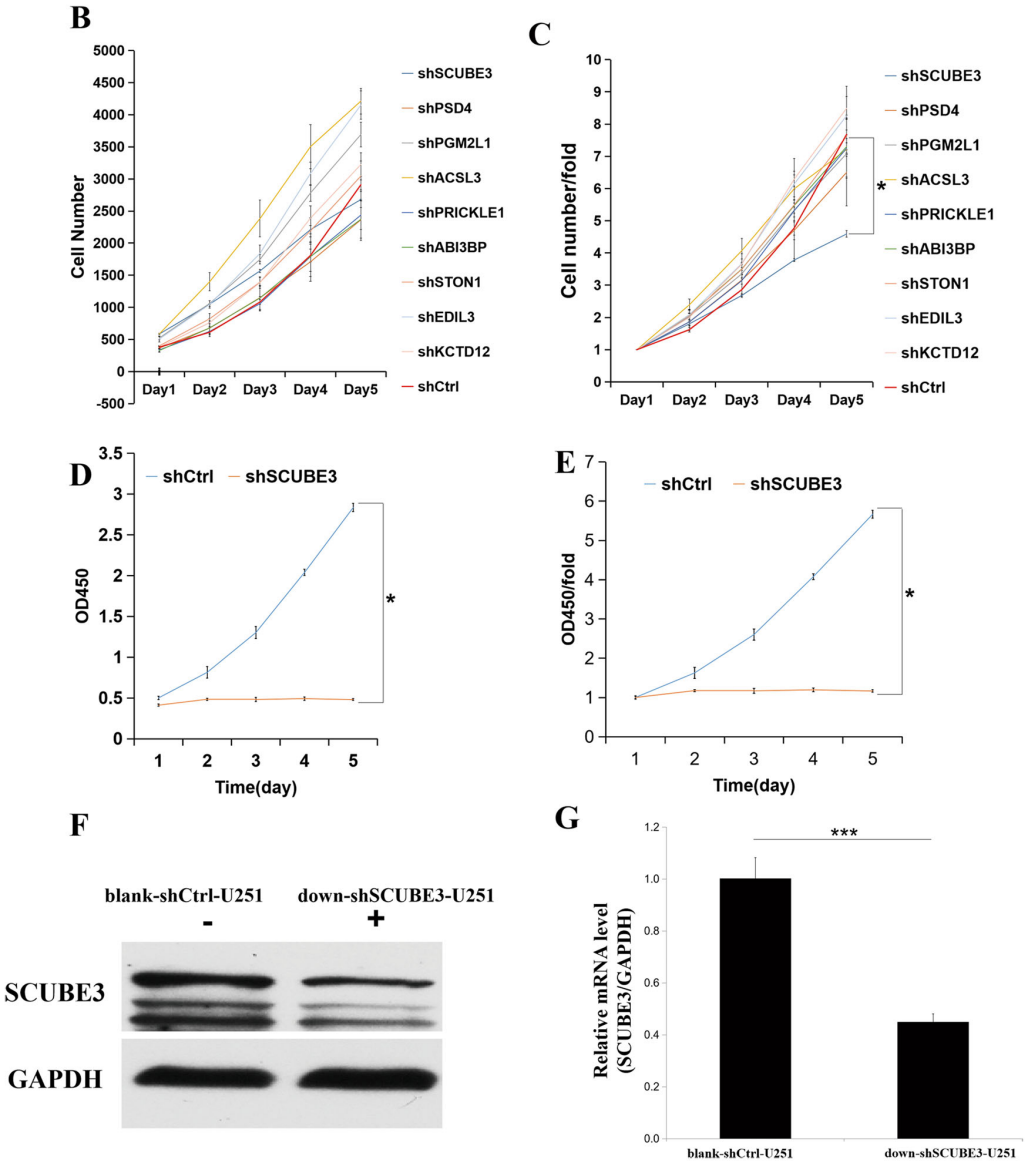
SCUBE3 silence inhibited U251 cell proliferation. **a** Effects of lentivirus-mediated silencing of nine genes in U251 cells as examined using fluorescence microscopy. The representative pictures shown are from one of three independent experiments; **b** 5 days of continuous counting of the cell numbers by HCS (*n* = 3); **c** 5 days of continuous counting of the cell numbers fold change by HCS (*n* = 3, *p < 0.05 compared to blank-shctrl U251); **d** 5 days of continuous CCK-8 assays (*n* = 3, *p < 0.05 compared with blank-shctrl U251); **e** 5 days of continuous CCK-8 assays, fold change (*n* = 3, *p < 0.05 compared with blank-shctrl U251); **f** the efficiency of knockdown SCUBE3 by shRNA was verified in U251 cells by western blot at 96 h (*n* = 3; “+” represents positive, and “−” represents negative compared with blank-shctrl U251); **g** the efficiency of knockdown SCUBE3 by shRNA was verified in U251 cells by qPCR at 96 h (*n* = 3, ***p < 0.001 compared with blank-shctrl U251).

**Fig. 4:**
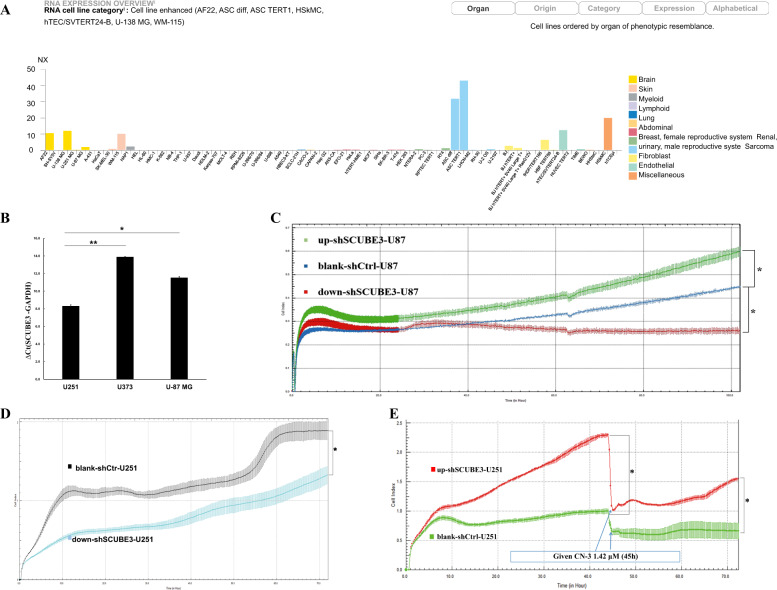
SCUBE3 is an oncogene in U251 and U87 cells. **a** Data in the Human Protein Atlas (https://www.proteinatlas.org/) showed SCUBE3 was enhanced in some cell lines; **b** SCUBE3 was highly expressed in U251 and U87 cells and showed moderate expression in U373 cells (∆Ct = SCUBE3 − GAPDH, if ∆Ct ≤ 12, then SCUBE3 was considered highly expressed; if 12 < ∆Ct < 16, then SCUBE3 was considered to show moderate expression; if ΔCt ≥ 16, then SCUBE3 was considered to show low expression, *n* = 3, *p < 0.05 and **p < 0.01 compared with ΔCt in U251); **c** iCELLigence showed knockdown of SCUBE3 inhibited U87 cell proliferation, and overexpression of SCUBE3 promoted U87 cell proliferation (*n* = 3, *p < 0.05 compared with blank-shctrl-U87); **d** iCELLigence showed knockdown of SCUBE3 inhibited U251 cell proliferation (*n* = 3, *p < 0.05 compared with blank-shctrl-U251); **e** iCELLigence showed overexpression of SCUBE3 promoted U251 cell proliferation and rescued the inhibition of U251 induced by CN-3 (*n* = 3, *p < 0.05 compared with blank-shctrl-U251).

U251 cells were transfected with shRNAs targeting the knockdown of nine genes (Fig. 3a). After 5 days, the blank-shctrl group of U251 cells showed growth of up to 7.68 times that of day 1 based on continuous cell counting by HCS. With regard to the targeted silencing of the nine genes, seven treatments resulted in growth below the level of the blank-shctrl group, i.e., 7.68 times (down-shSCUBE3, 4.59; down-shPSD4, 6.5; down-shPGM2L1, 7.08; down-shACSL3, 7.21; down-shPRICKLE1, 7.25; down-shABI3BP, 7.3; down-shSTON1, 7.63), while in two cases, growth was enhanced (down-shEDIL3, 8.26; down-shKCTD12, 8.49) (Fig. 3a–c). The down-shSCUBE3 transfection resulted in the most inhibition. Comparing the blank-shctrl group to the down-shSCUBE3 group on day 5, the proliferation rate fold change of the control group was up to 1.67 times higher (Fig. 3c), and the inhibition was supported by results of continuous CCK-8 assays conducted at the same time points (Fig. 3d, e). The efficiency of SCUBE3 knockdown in U251 cells was verified by western blot and qPCR (Fig. 3f, g). Based on data in the Human Protein Atlas (https://www.proteinatlas.org/), we observed that SCUBE3 expression was enhanced in some glioma cell lines (Fig. 4a). Because that the smaller the Ct value is, the less the number of cycles required for response amplification is, and the higher the initial content of the target gene is. Thus, ΔCt (SCUBE3 − GAPDH) revealed that the initial content of SCUBE3 is higher in U251 than it is in U87 or U373 (Fig. 4b). iCELLigence showed SCUBE3 is an oncogene in both U87 and U251 cells, with SCUBE3 knockdown resulting in inhibition of cell proliferation while SCUBE3 overexpression promotes their expression (Fig. 4c–e). Because SCUBE3 expression levels in the test glioma cell lines follows the trend U251 > U87 > U373 (Fig. 4b), and SCUBE3 overexpression rescued the inhibition of U251 induced by CN-3 (Fig. 4e), the U251 cell line was selected to for further experiments to determine the underlying mechanisms of CN-3 treatment.

### SCUBE3 knockdown may not induce caspase-dependent apoptosis in U251 cells

**Fig. 5:**
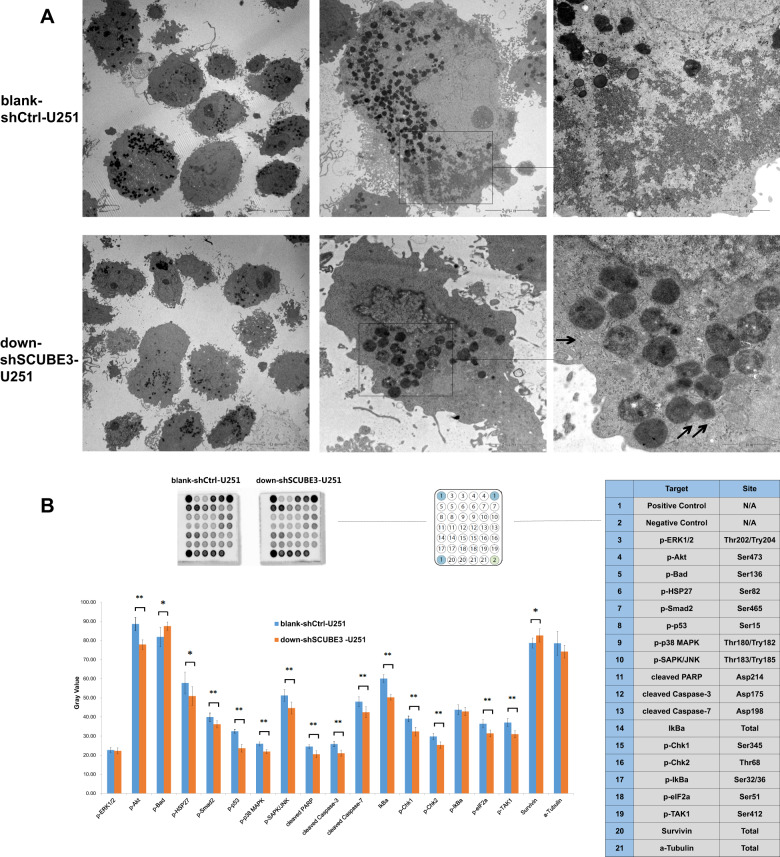
SCUBE3 knockdown may not induce caspase-dependent apoptosis in U251 cells. **a** Golgi swelling and endoplasmic reticulum swelling were observed in SCUBE3-knockdown U251 (pointed by arrows) under TEM; **b** a PathScan Stress and Apoptosis Signaling Antibody Array Kit was used for overall detection of 18 signaling molecules in down-shSCUBE3-transfected U251 cells. The expression level was calculated relative to the positive control signal as a gray value (*n* = 3, *p < 0.05, **p < 0.01 compared with blank-shctrl group).

Apoptosis and cell cycle arrest often contribute to decreased cell proliferation. TEM is often used to observe typical apoptotic morphology. We directly observed the down-shSCUBE3-U251 cells under TEM, and there was some Golgi swelling and endoplasmic reticulum swelling (as indicated by arrows) but not typical apoptosis (Fig. 5a). In caspase-dependent apoptosis, cleaved PARP, cleaved caspase-3, and cleaved caspse-7 are often increased, and survivin decreased. In the current study, a PathScan Stress and Apoptosis Signaling Antibody Array Kit was used for overall assessment of stress and apoptosis based on classical markers, but the results of treated cells did not correspond with a situation of caspase-dependent apoptosis (Fig. 5b). This indicates that SCUBE3 knockdown may not induce caspase-dependent apoptosis in U251 cells.

### SCUBE3 knockdown and CN-3 mediate Akt/p-Akt decrease to arrest U251 cells in G1/S transition

**Fig. 6:**
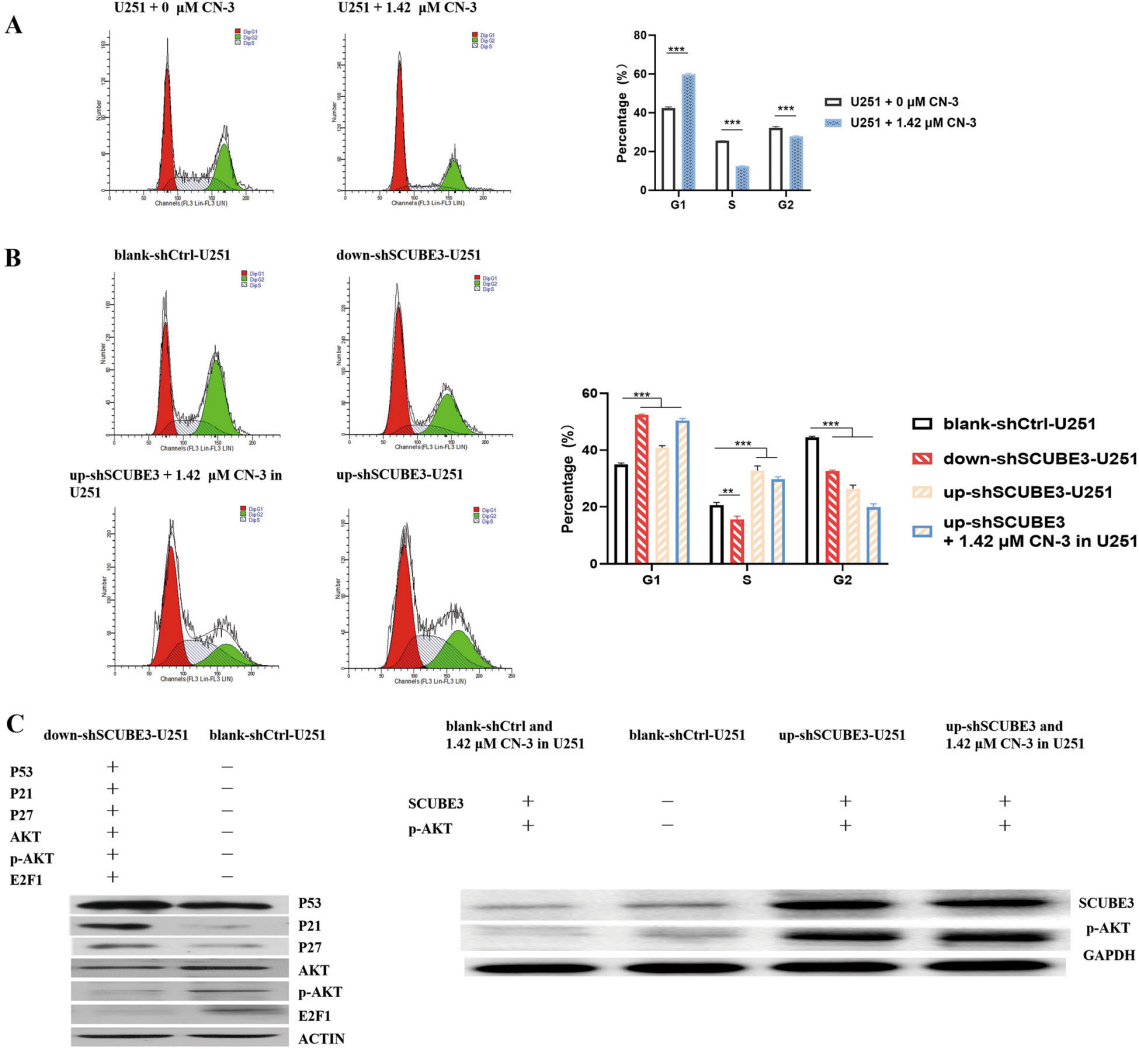
SCUBE3 knockdown arrested G1/S transition for CN-3 in U251 cells. **a** 1.42 μM CN-3 arrested G1/S transition in U251 cells examined by flow cytometry (*n* = 3, ***p < 0.001 compared with normal U251); **b** knockdown and overexpression of SCUBE3 revealed SCUBE3-mediated G1/S transition in U251 cells, and SCUBE3 overexpression rescued the arrest induced by CN-3 or SCUBE3 silencing (*n* = 3, **p < 0.01 and ***p < 0.001 compared with blank-shctrl group); **c** in U251 cells, western blot results showed knockdown of SCUBE3 increased p53, p27, and p21 and decreased Akt, p-Akt, and E2F1. Remarkably, SCUBE3 knockdown and 1.42 μM CN-3 decreased p-Akt in U251 cells, while overexpression of SCUBE3 abolished this decrease (*n* = 3; “+” represents positive, and “−” represents negative).

On the other hand, the detection of cell cycle by flow cytometry showed 1.42 μM CN-3 (Fig. 6a) and SCUBE3 knockdown arrested in U251 cells in G1/S transition (Fig. 6b), while SCUBE3 overexpression rescued the arrest induced by CN-3 or SCUBE3 silencing (Fig. 6b). The percentage of 1.42 μM CN-3-treated U251 cells in the G1 phase was significantly higher than that in normal untreated U251 (*p* < 0.001), while the percentage of cells in the S phase was significantly lower (*p* < 0.001) (Fig. 6a). The percentage of cells in the G1 phase in the down-shSCUBE3 group was significantly higher than that for the blank-shctrl group (*p* < 0.001), while the percentage of S phase cells was significantly lower (*p* < 0.01) (Fig. 6b). Comparing to the blank-shctrl group, overexpression of SCUBE3 significantly increased the proportion of U251 cells (*p* < 0.001) and CN-3-treated U251 cells (*p* < 0.001) in the S phase (Fig. 6b). At the molecular level, p53, p21, and p27 mediate cell cycle arrest while Akt, p-Akt, and E2F1 mediate cell cycle progression. SCUBE3 knockdown increased the levels of p53, p21, and p27 proteins and decreased the levels of Akt, p-Akt, and E2F1 proteins compared with blank-shctrl in U251 cells (Fig. 6c). Akt is activated primarily by phosphorylation. CN-3 (1.42 μM) reduced p-Akt and SCUBE3 in blank-shctrl U251 cells (Fig. 6c). The transfection with up-shSCUBE3 increased p-Akt and SCUBE3 in U251 and abolished the decrease of p-Akt and SCUBE3 induced by 1.42 μM CN-3 in U251 cells (Fig. 6c).

### SCUBE3 is highly expressed in glioma tissues from patients

**Fig. 7:**
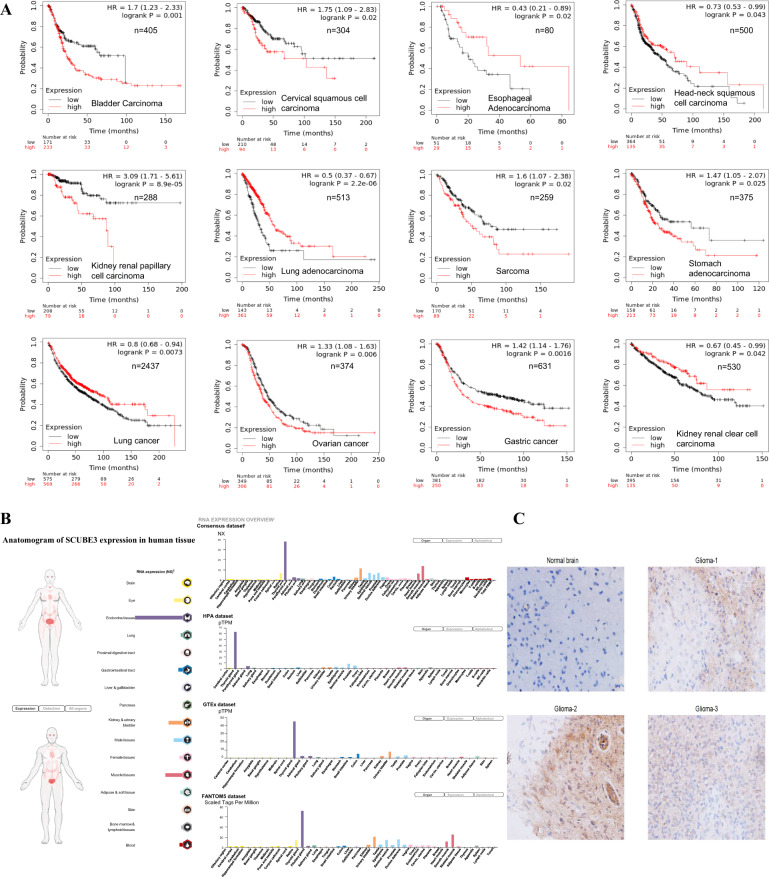
SCUBE3 protein was low expression in normal brain but enhanced in tumor specimens from glioma patients. **a** Kaplan–Meier survival curves (http://kmplot.com/analysis/) revealed when SCUBE3 was prognostic and favorable or unfavorable for the high and low expression group in pan-cancer analysis; **b** the Human Protein Atlas (https://www.proteinatlas.org/) was used to generate an anatomogram of SCUBE3 expression in human tissue, which pointed out that SCUBE3 was not highly expressed in brain; **c** immunohistochemical staining of SCUBE3 protein expression (brown area) in the normal brain and tumor specimens from glioma patients (*n* = 3, magnification 10 × 40).

Employing the Kaplan–Meier plotter^30^ (http://kmplot.com/analysis/), the Kaplan–Meier survival curves showed that SCUBE3 was prognostic and favorable in some cases (esophageal adenocarcinoma, head–neck squamous cell carcinoma, lung adenocarcinoma, lung cancer, and kidney renal clear cell carcinoma) and unfavorable in others (bladder carcinoma, cervical squamous cell carcinoma, kidney renal papillary cell carcinoma, sarcoma, stomach adenocarcinoma, ovarian cancer, and gastric cancer) when considering the high expression group and low expression group in pan-cancer analysis (Fig. 7a); this analysis did not include information on gliomas. Meanwhile, using the Human Protein Atlas (https://www.proteinatlas.org/), an anatomogram of SCUBE3 expression in human tissue pointed out that SCUBE3 was not highly expressed in the brain (Fig. 7b). To evaluate the relationship between SCUBE3 and gliomas, primary cancer specimens from three patients with histologically confirmed cases of glioma were examined immunohistochemically. This showed that SCUBE3 protein expression in tumors (indicated in brown) was obvious but very low in normal brain (Fig. 7c).

## Discussion

In recent years, the anticancer effect of saponins has been reported, including in our previous studies of saponins derived from some plants and animals^8–23^. In this paper, we isolated a new asterosaponin, CN-3, from the starfish *C. novaeguineae* (Table 1 and Fig. 1a) and found that CN-3 inhibited growth of U251 cells (IC_50_ = 1.59 μM, 48 h) (Fig. 1b) and U87 cells (IC_50_ = 1.418 μM, 48 h) (Fig. 1c). Interestingly, the other saponins we isolated from the starfish *C. novaeguineae* were not effective on glioma cells. This indicates that CN-3 may have antiglioma properties. The mechanisms of anticancer saponins involve numerous classical signaling pathways. It is well known that oncogenes have important roles in cancer. However, there have been very few studies reported on functional oncogene reduction induced by saponins in gliomas. In this study, 1.42 μM (IC_40_) of CN-3 suppressed U251 cell viability from 100 to 42.5% (24 h), 37.4% (48 h), and 52.1% (72 h) (Fig. 1d), while 1.34 μM (IC_40_) of CN-3 killed almost all U87 cells (48 h, 72 h) (Fig. 1e). Therefore, U251 cells treated with 1.42 μM CN-3 (48 h) were selected to carry out subsequent microarray experiments. Microarray analysis revealed that 661 genes had significantly differential expression (452 upregulated and 209 downregulated) in U251 following treatment with 1.42 μM CN-3 (Fig. 2a). From a pharmaceutical point of view, suppressing gene expression is more practicable than overexpression. Therefore, 9 out the 209 downregulation genes were selected, and their reduced expression was verified by qPCR (Fig. 2b). To discover potential functional oncogenes among the nine genes, U251 cells were transfected with targeted shRNA transfection of the nine genes. Five days of continuously counting the cell numbers by HCS showed that seven treatments resulted in reduced proliferation of U251 cells (Fig. 3a–c), with SCUBE3 knockdown resulted in the most inhibition. On day 5, comparing the blank-shctrl group to the down-shSCUBE3 group, the proliferation rate fold change was up to 1.67 times higher (Fig. 3c). The inhibition matched in the results of CCK-8 assays carried out at the same time (Fig. 3d, e). Further investigation using the Human Protein Atlas (https://www.proteinatlas.org/) revealed that SCUBE3 expression was enhanced in some glioma cell lines (Fig. 4a). Because that the smaller the Ct value is, the less the number of cycles required for response amplification is, and the higher the initial content of the target gene is. Thus, ΔCt (SCUBE3 − GAPDH) revealed that the initial content of SCUBE3 is higher in U251 than it is in U87 or U373 (Fig. 4b). iCELLigence tests showed SCUBE3 is an oncogene in both U251 and U87 cells, as SCUBE3 silencing reduced cell proliferation, whereas SCUBE3 overexpression promoted proliferation (Fig. 4c–e). Because the expression of SCUBE3 was highest in U251 among the test cell lines, and SCUBE3 overexpression rescued the inhibition of U251 induced by CN-3 (Fig. 4e), U251 was selected for the further mechanism experiments.

To determine the survival influenced by SCUBE3 knockdown in U251 cells, TEM was used to observe whether there were morphological changes in the down-shSCUBE3-transfected group. The TEM pictures showed there was some Golgi and endoplasmic reticulum swelling but no formation of any typical apoptotic bodies (Fig. 5a). Moreover, the PathScan Stress and Apoptosis Signaling Antibody Array Kit was used for overall detection of 18 signaling molecules that are involved in the regulation of the cellular stress response and apoptosis. With down-shSCUBE3 transfection, 16 signaling molecules significantly changed in U251 cells. Cleaved PARP, cleaved caspase-3, and cleaved caspse-7 are often increased, and survivin and p-BAD are decreased in caspase-dependent apoptosis. In the current study, the observations did not correspond with this tendency (Fig. 5b). Combining the results of TEM and the PathScan analysis, SCUBE3 knockdown does not appear to induce apoptosis in U251 cells (Fig. 5). On the other hand, detection of the cell cycle status by flow cytometry showed 1.42 μM CN-3 treatment arrested U251 cells in G1/S transition (Fig. 6a), and SCUBE3 knockdown had a similar effect (Fig. 6b). Overexpression of SCUBE3 canceled the G1/S arrest induced by SCUBE3 knockdown or 1.42 μM CN-3 in U251 cells by increasing the percentage S phase cells (Fig. 6b). Previous studies suggest that the molecular biochemical mechanisms underlying the anticancer activities of saponins are complex and work in a network fashion, and include phosphoinositide 3-kinase (PI3K)/protein kinase B (Akt)/mammalian target of rapamycin (mTOR) signaling pathway^9^, regulation of Fas and Bcl-2 family proteins^10^, NF-κB signaling pathway^12^, and BAD dephosphorylation and cleavage^14^. As a key signaling protein in the PI3K/Akt pathway, Akt is activated by phosphorylation and modulates a variety of downstream target proteins that are related to cell survival and proliferation^31^. Plumbagin, a natural naphthoquinone constituent isolated from the roots of medicinal plant *Plumbago zeylanica* L., reduced the level of p-Akt and suppressed the migration and invasion of U87 cells and U251 cells^32^. Mdm2, a ubiquitin ligase for p53, has a central role in regulation of the stability of p53 and serves as a good substrate for Akt. Decreased Akt activation in PRKD2-silenced cells could inactivate Mdm2 and thereby stabilize p53 in glioma cells. p53, p21, and p27 are known as mediators of cell cycle arrest. PRKD2 knockdown induced upregulation of p53, p21, and p27 expression. In a xenograft experiment, PRKD2 silencing significantly delayed tumor growth of U87 cells^33^. E2F1 promotes cell cycle progression from G1 to S phase, and the stability of cell cycle activator E2F1 affects the glioma cell proliferation^34^. Upregulation of E2F1 in astrocytoma and glioblastoma was associated with the progression of gliomas^35^, as indicated by direct binding of E2F1 to the pRb promotor in T98G glioma cells^36^. In the current study, knockdown of SCUBE3 increased p53, p21, and p27 and decreased Akt, p-Akt, and E2F1 in U251 cells (Fig. 6c). It is noteworthy that SCUBE3 knockdown and 1.42 μM CN-3 decreased p-Akt in U251 cells, while overexpression of SCUBE3 reversed the decrease both in 1.42 μM CN-3 or down-shSCUBE3-transfected U251 cells (Fig. 6c). This indicates that SCUBE3 mediated the inhibition for CN-3, and SCUBE3 knockdown arrested G1/S via Akt/p-Akt. In the examination of using PathScan, the decrease of p-p53 (Ser15), p-Chk1, p-Chk2, p-p38 MAPK, p-HSP27, p-SAPK/JNK, and p-TAK1 also support the finding that SCUBE3 knockdown suppresses U251 cell proliferation (Fig. 5b). Phosphorylation of p53 at Ser15, in response to DNA double-strand breaks, was due to ATM protein kinase^37–39^. Chk1 and Chk2 kinases are important proteins downstream of ATM/ATR in the DNA damage checkpoint control. Chk1 and Chk2 co-ordinate the DNA damage response and cell cycle checkpoint response by activating p-p53^40,41^. In breast cancer, KU60019 inhibited the p-p53 (Ser15), altered cell cycle checkpoints, and decreased DNA repair (via inhibiting the p-p53 and Ser15)^40^. In malignant glioma cells, shANKRD49 showed a decreased level of p-Chk1, indicating that Chk1 may participate in the oncogenic function of ANKRD49^42^. In breast cancer cell line MCF-7, a novel mTORC1/2 dual inhibitor INK128, which along with radiation downregulated p-Chk2, was combined with radiotherapy to significantly induce arrest in G2/M, double-strand breaks, and inhibition of their repair^43^. In this paper, p-p53 (Ser15), p-Chk1, and p-Chk2 were significantly decreased in U251 cells transfected with down-shSCUBE3 (*p* < 0.01) (Fig. 5b). HSP27 is a mediator of cell stress that confers resistance to adverse environmental conditions. It was reported that t-AUCB treatment induced Akt phosphorylation by activating Hsp27 in U251 and LN443 cell lines. Inhibition of Akt phosphorylation by Akt inhibitor IV sensitized glioblastoma cells to t-AUCB and strengthened t-AUCB in suppressing cell growth and inducing cell apoptosis, and inhibition of both p-Hsp27 and p-Akt could synergistically strengthen t-AUCB in suppressing cell growth^44^. p38 MAPK and SAPK/JNK MAP kinases are activated through a similar dual phosphorylation mechanism in response to pro-inflammatory cytokines and genotoxic stress. Cathepsin B and uPAR have key roles in cancer cell migration and invasion. Downregulation of uPAR and cathepsin B simultaneously caused the downregulation of phospho Akt, phospho p38 (MAPK), and PI3K and inhibit glioma cell migration^45^. A high-fat diet was observed to cause increased p-Akt, p-p38, and p-SAPK/JNK as well as increased angiogenesis, solid tumor growth, and lung metastasis of CT26 colon cancer cells^46^. Selective suppression of Notch1 inhibited proliferation of renal cell carcinoma cells through a reduction in p-SAPK/JNK and p-p38^47^. In the current study, the expression levels of p-p38 MAPK (*p* < 0.01), p-HSP27 (*p* < 0.05), and p-SAPK/JNK (*p* < 0.01) were significantly decreased by down-shSCUBE3 transfection of U251 cells (*p* < 0.01) (Fig. 5b). Smad2 is a key mediator of TGF-β signaling. Stimulation by TGF-β leads to Smad2 phosphorylation at Ser465/467 and translocation of Smad2 into the nucleus. The outcome of TGF-β signaling is context dependent and can either induce apoptosis or contribute to tumor cell metastasis^42^. In our study, the expression level of p-Smad2 was significantly decreased in U251 cells transfected with down-shSCUBE3 (*p* < 0.01). TAK1 is a kinase that can be activated by TGF-β, bone morphogenetic proteins, and other cytokines. In melanoma, epigallocatechin-3-gallate decreased p-TAK1 expression and suppressed cancer cell growth and metastasis by targeting TRAF6 activity^48^. In our study, the expression level of p-TAK1 was significantly decreased in U251 cells transfected with down-shSCUBE3 (*p* < 0.01) (Fig. 5b).

SCUBE3, a secreted and cell-surface EGF-CUB domain-containing protein, has been reported in a few cancers, specifically osteosarcoma^26^, lung cancer^27–29^, and salivary adenoid cystic carcinoma^49–51^. In osteosarcoma and lung cancer, SCUBE3 was highly expressed, and knockdown of SCUBE3 could inhibit the proliferation of cancer cells. The expression of SCUBE3 protein in osteosarcoma cells was significantly higher than that in normal osteocytes, and targeted inhibition of the gene could inhibit the proliferation of osteosarcoma^26^. SCUBE3 knockdown was associated with lower vascular permeability in the tumor and effectively decreased circulating tumor cells in the mice bearing SCUBE3-knockdown lung tumors^27^. SCUBE3 is an endogenous TGF-β receptor ligand, and knockdown of SCUBE3 expression also suppressed tumorigenesis and cancer metastasis in vivo in lung cancer^28^. Patients with high SCUBE3 expression had a significantly shorter survival time compared to patients with low SCUBE3 expression of non-small cell lung cancer^29^. However, the methylation marker of SCUBE3 was associated with poorer patient survival in renal cell carcinoma^49–51^. Kaplan–Meier survival curves^30^ (http://kmplot.com/analysis/) showed that SCUBE3 was prognostic and favorable in some cases (esophageal adenocarcinoma, head–neck squamous cell carcinoma, lung adenocarcinoma, lung cancer, and kidney renal clear cell carcinoma) or unfavorable in others (bladder carcinoma, cervical squamous cell carcinoma, kidney renal papillary cell carcinoma, sarcoma, stomach adenocarcinoma, ovarian cancer, and gastric cancer) when considering the high expression group and low expression group in pan-cancer analysis (Fig. 7a), but this did not include information on gliomas. There was little knowledge about SCUBE3 expression in gliomas. Using the Human Protein Atlas (https://www.proteinatlas.org/), we found that SCUBE3 was not highly expressed in the brain (Fig. 7b). Afterward, SCUBE3 protein was found to have high expression in primary glioma specimens from patients examined by immunohistochemistry but low expression in normal brain (Fig. 7c).

**Fig. 8:**
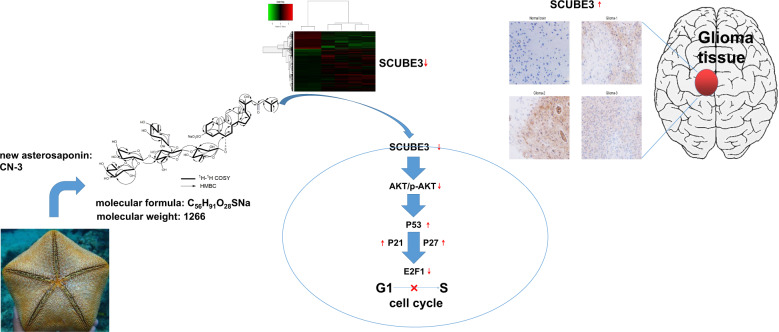
A schematic diagram of the molecular mechanism by which CN-3 arrests U251 cells in G1/S.

In conclusion, our study shows, for the first time, that SCUBE3 is an oncogene for cell proliferation in U251 and U87 cells, and that treatment with CN-3 isolated from starfish *C. novaeguineae* led to arrest of U251 cells in G1/S via the SCUBE3/ Akt/p-Akt/p53/p21/p27/E2F1 pathway (Fig. 8). These findings provide evidence and novel insights for the development of CN-3 into a novel candidate for the treatment of gliomas. Although this study provided some clues between SCUBE3 and CN-3 antiglioma, there are still some important and interesting questions that should be answered in the near future. According to the microarray analysis, treatment with a low concentration of CN-3 significantly induced differential expression in the case of hundreds of genes, and this is related to its antiglioma effect. Were there more new functional genes participating in the antiglioma effect of CN-3? To understand the function of genes like SCUBE3, their role should be examined in more glioma cell lines both in *vitro* and in vivo. Furthermore, secreted and/or cell-surface proteins like SCUBE3, in particular, should be examined in more glioma clinical samples, including tissue specimens, cerebrospinal fluid, and blood. At the bottom of the anatomogram of SCUBE3 expression in human tissue, we noticed that SCUBE3 was expressed in blood (Fig. 7b).

## Materials and methods

See “Supplementary Materials and methods” section for additional details.

### Extraction and isolation

The starfish *C. novaeguineae* (100 kg, wet weight) was cut into pieces and then extracted with 75% ethanol three times, each time for 2 h, under reflux. The extract was combined and dried in vacuo to give a residue, which was suspended in water and then partitioned with petroleum ether and *n*-butanol sequentially. The *n*-butanol part (320 g) was subjected to a silica gel column chromatography eluting with a CHCl_3_/CH_3_OH/H_2_O (50:1:0 to 6:3.5:1) gradient to give 16 major fractions. Fraction 13 (20 g) was subjected to size exclusion chromatography on a Sephadex LH-20 column equilibrated with CHCl_3_/CH_3_OH (1:1) to remove the impurities and give five subfractions (Fr.13-1 to Fr.13-5). Fraction 13-2 was chromatographed on a C18 reversed-phase column eluting with CH_3_OH/H_2_O (2:3 to 1:0) to give two subfractions (Fr.13-2-1 and Fr.13-2-2). CN-3 (160 MG cells, tR = 32.5 min) was obtained from Fr.13-2-1 by HPLC eluting with CH_3_CN/H_2_O (1:1) at a flow rate of 6 mL/min. Then the asterosaponin was dissolved in 5% DMSO as 7 MG cells/mL (5.529 mM) and stored at 4 °C.

### Gene expression microarray analysis

The R Bioconductor affy packag ENREF RMA (robust multi-array average) function was used to compute expression. The CEL files and RMA normalized expression values can be downloaded from https://www.ncbi.nlm.nih.gov/geo/query/acc.cgi?acc=GSE108343. Affymetrix GeneChip PrimeView Human Gene Expression Array probe annotations were based on NetAffx gene annotation symbols.

### Cell culture and lentiviral infection

Human glioma cell lines U251, U87, U373, and human embryonic kidney cell line 293T were obtained from the Cell Bank of Chinese Academy of Science (Shanghai, China). The source of cell lines and report was authenticated by Shanghai Genechem (Shanghai, China). The cell lines were cultured in DMEM (Corning) supplemented with 10% FBS (Ausbian) at 37 °C with 5% CO_2_. The cell lines were randomly divided into two groups, namely treated and untreated. U251 and U87 cells were randomly divided into two groups, namely blank-shctrl groups (infected with blank viral), and shSCUBE3 groups (infected with interference lentivirus: down-shSCUBE3 represented SCUBE3 knockdown and up-shSCUBE3 represented SCUBE3 over expression). The lentiviral infection efficiency was observed using a fluorescence microscope after cultured for 3 days.

### Patient and tumor specimens

Glioma tumor tissue from patients with histologically confirmed glioma who underwent surgical resection and normal tissue specimens used for immunohistochemical staining were obtained at the Xijing Hospital (Xi’an, Shaanxi, China). None of the patients had received preoperative adjuvant chemotherapy or radiation therapy.

### Statistical analysis

Experiments were performed in biological replicates, most of which included at minimum technical triplicates (*n* = 3), as indicated in the figure legends. Sample size depended on the assay type. The investigators were blinded to the group allocation during the experiment when assessing the outcome of qPCR, western blot, transmission electron microscope (TEM), immunohistochemistry, pathscan, and flow cytometer detection. For all groups that are statistically compared, the variance within each group was similar. Means were compared with one-way analysis of variance or two-way analysis of variance when applicable. Multiple comparisons were performed by Tukey’s multiple comparisons test. All data are presented as the mean ± standard deviation (SD) and analyzed using GraphPad Prism. A value of *p* < 0.05 was considered as statistically significant difference.

## Supplementary Information

Supplementary Information
