# Supplementary material for: Reduction of SCUBE3 by a new marine-derived asterosaponin leads to arrest of glioma cells in G1/S
Source: Oncogenesis. 2020 Aug 6;9(8):71. doi: 10.1038/s41389-020-00252-4 (PMC7411020; doi:10.1038/s41389-020-00252-4)
Supplement: Supplementary file 1 — Supplementary Information [file 41389_2020_252_MOESM1_ESM.doc]

Supplementary Information for

Reduction of SCUBE3 by a New Marine-Derived Asterosaponin Leads to Arrest of Glioma Cells in G1/S

Peng-Cheng Qiu ^1, †^, Yun-Yang Lu ^1, †^, Shan Zhang ^1^,**^2^**, Hua Li ^1^, Han Bao ^1^, Yu-Qiang Ji ^3^, Fei Fang ^3^, Hai-Feng Tang ^1^, *& Guang Cheng ^4^, *

^1^ Institute of Materia Medica, Key Laboratory of Gastrointestinal Pharmacology of Chinese Materia Medica of the State Administration of Traditional Chinese Medicine, School of Pharmacy, Air Force Medical University, Xi'an 710032, P.R. China; [qpc023@126.com](mailto:qpc023@126.com) (P.-C.Q.); [luyunyanggq@163.com](mailto:luyunyanggq@163.com) (Y.-Y.L.); [574481003@qq.com](mailto:574481003@qq.com) (S.Z.); [lihuasmile@aliyun.com](mailto:lihuasmile@aliyun.com) (H.L.); [bmds321@163.com](mailto:bmds321@163.com) (H.B.); [tanghaifeng71@163.com](mailto:tanghaifeng71@163.com) (H.-F.T.).

^2^ School of Pharmacy, Shaanxi University of Chinese Medicine, Xianyang 712046, P.R. China.

^3^ Central Laboratory of Xi'an No.1 hospital, Xi'an, 710002, P.R. China; [jiyuqiang112299@126.com](mailto:jiyuqiang112299@126.com) (Y.-Q.J.); [307555806@qq.com](mailto:307555806@qq.com) (F.F.).

^4^ Department of Neurosurgery, Xijing Institute of Clinical Neuroscience, Air Force Medical University, Xi'an, 710032, P.R. China. [chg16801@163.com](mailto:chg16801@163.com) (G.C.)

**†** These authors contributed equally to this work.

Short Title: Down-SCUBE3 by CN-3 leads to glioma G1/S arrest

* Corresponding Author

Hai-Feng Tang

Institute of Materia Medica, Key Laboratory of Gastrointestinal Pharmacology of Chinese Materia Medica of the State Administration of Traditional Chinese Medicine, School of Pharmacy, Air Force Medical University, Xi'an 710032, P.R. China.

Tel: 0086-29-84774748

FAX: 0086-29-83224790

E-mail: [tanghaifeng71@163.com](mailto:tanghaifeng71@163.com)

Guang Cheng

Department of Neurosurgery, Xijing Institute of Clinical Neuroscience, Air Force Military Medical University, Xi'an, 710032, P.R. China.

Tel: 0086-29-84775328

FAX: 0086-29-83224790

1. mail: [chg16801@163.com](mailto:chg16801@163.com)

This PDF file includes:

Structure Elucidation

Supplementary Materials and Methods

References

**Structure** **Elucidation**

The new asterosaponin CN-3 was isolated from starfish *Culcita novaeguineae.* The molecular weight was 1266 and molecular formula was determined as C_56_H_91_NaO_28_S, from ESI-MS *m/z* 1289 [M+Na]^+^ and HR-ESI-MS *m/z* 1289.5206 [M+Na]^+^(calcd for C_56_H_91_Na_2_O_28_S, 1289.5213). The fragment ion peak at *m/z* 1169 [M+Na-NaHSO_4_]^+^ in the positive ion mode ESI-MS indicated the presence of a sulfate group in the glycoside. This was confirmed by the IR spectrum with absorption bands at 1242 and 1213 cm^-1^. A comprehensive analysis of the ^1^H-NMR (500 MHz) and ^13^C-NMR (125 MHz) spectra of asterosaponin CN-3 suggested the presence of a steroidal aglycone with one methine group (*δ*_H_ 4.83, *δ*_C_ 77.6, C-3), associated with an *O*-sulfate group, one oxygenated methine (*δ*_H_ 3.67, *δ*_C_ 80.5, C-6), the trisubstituted 9(11)-double bond (*δ*_H_ 5.13, *δ*_C_ 116.7, 145.5), two angular methyl group (*δ*_H_ 0.90, *δ*_C_ 13.5, C-18; *δ*_H_ 0.84, *δ*_C_ 19.3, C-19), one carbon bearing a hydroxy group (*δ*_C_ 73.7, C-20) and one ketone carbenyl group (*δ*_C_ 211.5, C-23). Detailed analysis of the 2D NMR (HSQC, COSY, HMBC) data led to the assignment of all the proton and carbon resonances of asterosaponin CN-3 (table 1). The NOESY correlation of H-3 (*δ*_H_ 4.83) to H-5 (*δ*_H_ 1.35) and H-14 (*δ*_H_ 1.18) to H-17 (*δ*_H_ 1.55) and H-21 (*δ*_H_ 1.46) indicated the *α*-orientation of H-3, H-17 and H-21. The β-orientation of H-6 was deduced from the NOESY correlation of H-6 (*δ*_H_ 3.67) to H-8 (*δ*_H_ 1.97) and H-19(*δ*_H_ 0.84). Therefore, the aglycone of asterosaponin CN-3 was identified as (20*S*)-5*α*-cholest-9(11)-en-23-one-3*β*,6*α*,20-triol 3-sulfate which was the same as thornasterol A and novaeguinoside B. The sugar moieties of asterosaponin CN-3 were determined to be D-quinovose, D-glucose, D-fucose and L-arabinose in a ratio 2:1:1:1 by acidic hydrolysis followed by derivatization and HPLC analysis. In addition, the ^13^C-NMR spectrum of CN-3 revealed five anomeric carbon signals at *δ*_C_ 105.1 (Qui I), *δ*_C_ 103.9 (Glc), *δ*_C_ 105.0 (Qui II), *δ*_C_ 101.3 (Ara) and *δ*_C_ 106.6 (Fuc), which correlated with the corresponding anomeric protons at *δ*_H_ 4.68 (d, 7.2), *δ*_H_ 4.98 (d, 7.8), *δ*_H_ 5.14 (d, 6.4), *δ*_H_ 4.84 (d, 7.2) and *δ*_H_ 4.72 (d, 7.8) in the HSQC spectrum, confirming the presence of five sugar moieties. The coupling constants (6.4-7.8 Hz) of the anomeric protons indicated a *trans*-diaxial orientation with respect of their coupling partners (*β*-configuration for D-quinovose, D-glucose, D-fucose, and *α*-configuration for L-arabinose). The assignments of the signals attributable to various sugar units were achieved by the application of 2D NMR experiments including ^1^H-^1^H COSY, HMBC, TOCSY and NOESY techniques (table 1). The sequence of oligosaccharide moiety was confirmed by analysis of HMBC spectrum of CN-3. The terminal fucose at the C-2 of Ara was deduced from the cross peak *δ*_H_ 4.72 (Fuc, H-1) to *δ*_C_ 81.7 (Ara, C-2). The arabinose at the C-4 of Glc and the terminal quinovose (Qui II) at the C-2 of Glc were indicated by the cross peaks *δ*_H_ 4.84 (Ara, H-1) to *δ*_C_ 80.3 (Glc, C-4) and *δ*_H_ 5.14 (Qui II, H-1) to *δ*_C_ 81.3 (Glc, C-2). The HMBC correlation of *δ*_H_ 4.98 (Glc, H-1) to *δ*_C_ 90.4 (Qui I, C-3) suggested the linkage of glucose to C-3 of Qui I. The glycosidation at C-6 of the aglycone was indicated by HMBC correlation of *δ*_H_ 4.68 (Qui I, H-1) to *δ*_C_ 80.5 (aglycone, C-6). The linkage of oligosaccharide moiety was in coincidence with the correlations between H-6 of aglycone and Qui I H-1, between H-1 of Glu and Qui I H-3, between H-1 of Qui II and Glc H-2, between H-1 of Ara and Glc H-4, between H-1 of Fuc and Ara H-2, in the NOESY spectrum. Consequently, the structure of the asterosaponin CN-3 was elucidated as sodium (20*S*)-6*α*-*O*-{*β*-D-fucopyranosyl-(1-2)-*α*-L-arabinopyranosyl-(1-4)-[*β*-D-quinovopyranosyl-(1-2)]-*β*-D-glucopyranosyl-(1-3)-*β*-D-quinovopyranosyl}-20-hydroxy-23-oxo-5*α*-cholest-9(11)-en-3*β*-yl sulfate.

Supplementary Materials and Methods

Plasmid Construction and Lentivirus Packaging

To create SCUBE3 shRNA-silenced sub cell lines, we have used the following shRNA sequences designed against SCUBE3 gene (NM_152753): 5’-CCGGGCAGAGCTGTGTCAACATGATCTCGAGATCATGTTGACACAGCTCTGCTTTTTG-3’ (S1) and 5’-AATTCAAAAAGCAGAGCTGTGTCAACATGATCTCGAGATCATGTTGACACAGCTCTGC-3’ (S2). Short hairpin RNA (shRNA) expression vector GV115, and helper plasmids helper 1.0 and helper 2.0 were purchased from Shanghai Genechem (Shanghai, China). The lentiviral particles were constructed according to previous report^1^. The plasmids were then transfected into 293T cell using Lipofectamine 2000 (Life Technologies, Grand Island, NY, USA). After 24 h, the transfection efficiency was assessed by observing the expression of fluorescence-marked gene (GFP). Once transfection efficiency was more than 70%, the cells were harvested after 36 h to screen effective RNAi vector using western blotting assay. As a result, the effective fragment was as follow: 5’-CCGGGCAGAGCTGTGTCAACATGATCTCGAGATCATGTTGACACAGCTCTGCTTTTTG-3’. The effective interference lentivirus vectors were transfected into 293T cell combined with packaging plasmids. After 48 h, the supernatant containing lentiviral particles was harvested and centrifuged at the speed of 4,000 × g for 10 min. And then the lentivirus was filtered using 0.45 μm cellulose acetate filters and stored at -80 ℃ until use. The lentivirus were purchased from Shanghai Genechem (Shanghai, China): ShRNA-targeting PGM2L1, batch number psc26567mix; ShRNA-targeting EDIL3, batch number psc26963mix; ShRNA-targeting SCUBE3, batch number psc33608mix; ShRNA-targeting PRICKLE1, batch number psc57144mix; ShRNA-targeting KCTD12, batch number psc48274mix; ShRNA-targeting ACSL3, batch number psc52774mix; ShRNA-targeting PSD4, batch number psc53510mix; ShRNA-targeting ABI3BP, batch number psc57126mix; ShRNA-targeting STON1, batch number psc57099mix.

High-content screening assay

U251 cells infected with blank-shcrl lentivirus or down-shSCUBE3 lentivirus were seeded and cultured in 96-well plates for 5 days. Cell numbers in each well was determined using the ArrayScanTM high-content screening (HCS) software (Cellomics Inc) each day. The number and distribution of stained cells were identified and analyzed by the fluorescence imaging microscope. Images were acquired using appropriate filters with 20× objective and stored in a microsoft structured query language (SQL) database.

Real Time Cellular Analysis (RTCA)

The proliferation assay and the cell growth index were recorded using iCELLigence system (ACEA Biosciences, Inc., San Diego, CA, USA) as the RTCA system. All monitoring was performed at 37 ℃ with 5% CO_2_. E-plates (culture plates for the iCELLigence system) containing 300 µl culture medium per well were equilibrated to 37 ℃, the cells were seeded at 1×10^4^ cells per well in cell culture.

Stress and apoptosis signaling assay

To address the signaling pathway involved in the phenotype induced by down-shSCUBE3, we detected the modifications in a set of cellular proteins playing a well-understood role in cell proliferation and apoptosis using pathscan® stress and apoptosis signaling array kit (Cell Signaling Technology, #12856). U251 lysate was prepared and detected according to the protocol provided by CST. The intensity of each signal was measured by Image J software.

Flow Cytometric Analysis of Cell Cycle

Before flow cytometric detection, cells were cultured overnight in DMEM supplemented without FBS at 37 ℃ with 5% CO_2_. Then the groups were again cultured in DMEM supplemented with 10% FBS at 37 ℃ with 5% CO_2_ for no more than 4 h. Flow cytometric analyses of cell cycle were performed with Annexin V staining kit (Roche, USA), according to the manufacturer’s instruction.

Quantitative Real-time PCR analysis

qPCR analysis was performed using the SYBR Green Master Mix Kit in the DNA Engine Opticon™ System (MJ Research, Waltham, MA). GAPDH was used as an internal control. The primers of SCUBE3 were 5′-GCTGTGTCAACATGATGGGC-3′ (forward) and 5′-CCGCTGGATACAGGTATGCTG-3′ (reverse). The primers of other genes were shown in table 2. The relative gene expression levels were calculated and statistically compared using the 2-ΔΔCT analysis program.

Western Blot

Cells were washed with phosphate balanced solution (PBS) and transferred into a 1.5 ml EP tube. Then the cells were lysed using lysis buffer on ice for 10-15 min and then centrifuged at 4 ℃, 12000 × g for 15 min. The concentration of protein was detected and adjusted to a final concentration of 2 µg/µl. After mixed with an equal volume of 2 × loading buffer, the protein samples were denatured at 100 ℃ for 5 min. The protein samples were separated using 10% SDS-polyacrylamide gels (SDSPAGE) and transferred onto a polyvinylidene fluoride (PVDF) membrane. After blocked with 5% skim milk for 1 h, anti-SCUBE3 (BIOSS Inc., China, product code: 12317R), anti-p21 (Wuhan-Sanying Inc., China, product code: 10355-AP), anti-p27 (Wuhan-Sanying Inc., China, product code: 25614-1-AP), anti-p53 (Affinity Biosciences, Inc., USA, product code: AF0879), anti-Akt (Affinity Biosciences, Inc., USA, product code: AF3074), anti-pAkt (Affinity Biosciences, Inc., USA, product code: AF0908), anti-βactin (Servicebio Inc., China, product code: GB12001), and anti-GAPDH (Santa Cruz Biotechnology, Inc., USA, product code: sc-32233) were added as primary antibodies for the specific protein for 2 h at room temperature. The immunoreactive bands were detected and visualized by electrochemiluminescent (ECL) detection system (Amersham Life Sciences, Inc., Arlington Heights, IL, USA). All the experiments were conducted for three times in several days.

References

1 Tang J. et al. Metastasis associated in colon cancer 1 (MACC1) promotes growth and metastasis processes of colon cancer cells. Eur Rev Med Pharmacol Sci.20, 2825-2834 (2016).
